# Nutritional interventions in adult fibrostenotic Crohn’s disease: A systematic review

**DOI:** 10.3389/fnut.2023.1017382

**Published:** 2023-02-21

**Authors:** Jared L. Cooper, Ryan E. Rosentreter, Alexis Filyk, Zahra A. Premji, Hua Shen, Richard Ingram, Gilaad G. Kaplan, Christopher Ma, Kerri Novak, Remo Panaccione, Cynthia H. Seow, Florian Rieder, Maitreyi Raman, Cathy Lu

**Affiliations:** ^1^Department of Medicine, University of Calgary, Calgary, AB, Canada; ^2^Division of Gastroenterology and Hepatology, Department of Medicine, University of Calgary, Calgary, AB, Canada; ^3^Libraries, University of Victoria, Victoria, BC, Canada; ^4^Department of Mathematics and Statistics, University of Calgary, Calgary, AB, Canada; ^5^Department of Community Health Sciences, University of Calgary, Calgary, AB, Canada; ^6^Department of Inflammation and Immunity, Lerner Research Institute, Cleveland Clinic Foundation, Cleveland, OH, United States; ^7^Department of Gastroenterology, Hepatology and Nutrition, Digestive Diseases and Surgery Institute, Cleveland Clinic Foundation, Cleveland, OH, United States

**Keywords:** Crohn’s disease, fibrostenosis, stricture, diet, nutrition

## Abstract

**Background:**

Management of Crohn’s disease (CD) using dietary interventions has become an area of increased research interest. There is a lack of specific research exploring if diet and nutrition interventions are beneficial in patients with strictures, as current dietary recommendations in fibrostenotic CD are often based on clinical judgment. The aim of this systematic review was to assess the impact of dietary interventions in fibrostenotic CD on medical and surgical outcomes.

**Methods:**

A systematic search of MEDLINE (Ovid), EMBASE (Ovid), CINAHL (EBSCO), and Cochrane Central Register of Controlled Trials (Ovid) was conducted. Studies reporting dietary interventions or nutritional factors in fibrostenotic CD were included. Outcomes for studies assessing dietary interventions such as enteral nutrition were evaluated as changes in (1) CD symptoms (CD Activity Index), (2) stricture parameters on diagnostic imaging, and (3) rates of surgical or medical intervention following dietary interventions.

**Results:**

Five studies were included in this review. Three studies assessed exclusive enteral nutrition (EEN), one evaluated total parenteral nutrition (TPN), and one studied a liquid diet. All included studies evaluated symptoms as an outcome, while diagnostic imaging parameters and surgical outcomes in the studies were either absent or too heterogeneous to appraise improvement post dietary intervention. Included EEN studies displayed similar efficacy, with approximately 60% of patients having symptom improvement. The included TPN study also reported 75% of patients with symptom improvement, while the liquid diet did not.

**Conclusion:**

Exclusive enteral nutrition and total parental nutrition may provide benefit for use as a dietary intervention for fibrostenotic CD. There remains a need for high-quality controlled trials which utilize standardized definitions of strictures.

## Introduction

Crohn’s disease (CD) is an inflammatory bowel disease (IBD) that can frequently develop stricturing complications anywhere along the gastrointestinal tract. Strictures occur most commonly at the small intestine, and these patients may present with obstructive symptoms that require medical management including corticosteroids, immunomodulators, biologics (1), and/or endoscopic interventions with balloon dilation (2, 3), and surgical management (4). It is estimated that over 50% of patients with CD will develop either a stricture or penetrating complication within 20 years of diagnosis (5). The approach and treatment of patients with stricturing CD is a challenge as currently no specific anti-fibrotic medications are available. The diagnosis of stricturing CD is dependent on symptoms, laboratory markers, endoscopy, and diagnostic imaging. Although there are classic obstructive symptoms such as abdominal pain, post-prandial bloating, and nausea and/or vomiting, it is well known that symptoms do not always correlate with stricture extent and severity, and as a result, cross-sectional imaging plays a pivotal role in the diagnosis and monitoring of strictures (6). Consensus definitions of small bowel strictures on computed tomography (CT) and magnetic resonance enterography (MRE) have been developed (7). Features of strictures that are included in this definition include bowel wall thickness (BWT), luminal apposition, and pre-stenotic dilation for both naïve and anastomotic strictures. Furthermore, intestinal ultrasound (IUS) is a non-invasive, accurate, and low-cost imaging modality with widespread use in Europe, and rapidly growing use in North America. A program to build a consensus definition of small bowel strictures on IUS is currently in development.

Over the past decade, the role of diet and nutritional management in CD has garnered increasing research interest due to the recognition of the impact that malnutrition has on disease course and activity, as well as its potential as a therapeutic intervention (8–10). The prevalence of malnutrition in CD is estimated at 17% (11), with patients at risk of both micro and macronutrient deficiencies (12, 13). Furthermore, diet and nutrition are of great concern to the patients themselves with 89.7% avoiding certain food groups for fear of worsening or inciting a disease flare, and > 85% of CD patients reporting that they believe dietary advice from a qualified professional would be useful (11). It has been increasingly recognized that patients with fibrostenotic CD are at a higher risk for nutritional deficits due to the avoidance of certain foods that may exacerbate symptoms, as well as altered absorption in those with previous bowel resections (14). Dietary interventions have the capacity to alter the disease course in CD through increases in microbiome diversity and alterations of metabolism, immune cell interactions, as well as epigenetic modulation (15, 16). However, despite this increased emphasis on diet and nutrition in the setting of CD, research into the efficacy of dietary interventions within distinct phenotypes such as the stricturing subtype is limited. Many of the current nutritional recommendations in the management of CD are based on expert opinion only (17), and do not stratify nutritional interventions based on the heterogeneous CD phenotypes.

In this systematic review, we aim to address this knowledge gap by providing a comprehensive summary of nutritional interventions such as enteral and parenteral nutrition in established CD strictures to assess its impact on symptom improvement, surgical intervention, alterations in CD medications, and diagnostic imaging findings.

## Methods

### Search strategy and study selection

A systematic literature review was performed according to the Cochrane guidelines (18), and is reported below according to the PRISMA guidelines (19).

Medline (Ovid), EMBASE (Ovid), Cochrane Central Register of Controlled Trials (Ovid) and CINAHL (EBSCO) were searched to locate available research from the first available date until March 27, 2022. The database search strategies were created with the assistance of a medical librarian. The search strategy was comprised of three search concepts: Crohn’s disease, strictures, and dietary factors. Each search concept included controlled vocabulary, where available, and keywords searched within the title, abstract, and keywords fields. Individual search lines were combined using appropriate Boolean operators. The exact line-by-line search strategies for all database searches have been included in Supplementary Tables S3–S6. Records from the searches were exported from each database as RIS files and imported into Covidence for deduplication and screening. The reference lists of studies included after screening were reviewed manually to identify additional relevant publications. A review protocol describing the rationale, hypothesis and planned methods of the review was prepared for internal purposes prior to initiating this systematic review.

### Inclusion/exclusion criteria

Studies were considered eligible if the following criteria was met: (1) study design was either interventional design (randomized or non-randomized), or observational design (prospective or retrospective) and either case–control, case series, cross-sectional studies, or cohort studies, (2) human population, (3) adult population (age ≥ 18 years), (4) simple size greater than 3, and (5) CD stricture diagnosed on full thickness histopathology, diagnostic imaging, or endoscopy. Exclusion criteria were as follows: (1) non-human population studied, (2) narrative reviews, and (3) articles published in languages other than English.

### Outcomes of interest

The outcomes of interest used in this review for studies where a dietary intervention was used for patients with fibrostenotic CD were rates of improvement in CDAI, surgical interventions, and any changes in diagnostic imaging pre and post dietary interventions.

### Study selection and data extraction

Two Reviewers (J.C, A.F) independently screened titles and abstracts using Covidence software in accordance with the eligibility criteria. The full text of articles remaining after title/abstract screening were retrieved and subsequently independently reviewed by two reviewers based on the eligibility criteria. Cases without consensus between reviewers were resolved by consultation of a third-party expert (C.L) for a final decision.

Two reviewers (J.C, A.F) independently extracted data from each study including: first author name, journal, year of publication, country where study was conducted, study design, CD stricture definition, diagnostic modality for CD stricture diagnosis, total number of patients, number of patients with confirmed CD strictures often as a subgroup of a larger study, dietary interventions, nutrition related risk factors (weight, body mass index, malnutrition), primary end point investigated, follow up period, response to nutritional intervention, and complications. Table 1 summarizes the extracted data variables.

**Table 1:** Summary of study details from literature review of dietary interventions in fibrostenotic Crohn’s disease.

First Author, publication year	Country	Study design	Stricture definition	Diagnostic modality of stricture (clinical, DI, endoscopy)	Number of CD patients	Number of patients with strictures	Dietary intervention	Aim of diet therapy	Nutrition related risk factor (weight, BMI, malnutrition)	Follow up period	Primary endpoint investigated (surgical rates, DI findings, med changes, etc.)	Adherence of therapy described (Y/N)	Reported adherence	Outcomes		Complications
														Disease activity markers	Symptoms	
Hu, 2014	China	P	Elevated acute inflammatory variables, radiologic string sign, thickened bowel wall on CT	Clinical assessment, radiography, CT and endoscopy	50	50	EEN elemental formula Peptide: Peptisorb. Oral suspension during day and NGF at night. Diet given for 12 weeks	Relieve inflammatory bowel strictures of CD	N/A	N/A	CR, SR, and RR. SR was defined as a decrease of symptomatic degree from high level to a lower one. RR was defined as a 100% increase of bowel luminal cross-sectional area after treatment. CR was defined as a > 70-point decrease of CDAI score. Complete remission required that the patients simultaneously fulfilled criteria for SR, RR, and CR.	Yes, self-reported/telephone interview	59/65	Only 13.8% displayed progressive bowel obstruction resulting in bowel resection. Of these (*n* = 9), 4 were noted as having fibrotic strictures post-surgery. 46.1% achieved complete remission (64.6% CR, 53.8% RR). 59% decrease of bowel wall thickness with a 331% increase of luminal cross-sectional area at week 12 compared with baseline. CRP: 63% decrease Albumin: 29% increase	73.8% SR	26.2% had diarrhea and/or abdominal distention due to inadequate infusion of home EN. This was mitigated by physician correction.
Yang, 2017	China	P	Lennard-Jones JE Classification of inflammatory bowel disease.	CT enterography or MR enterography and endoscopy. Patients with stricture also underwent enhanced ultrasound to further differentiate inflammation from fibrosis.	41	10	EEN for 12 weeks, NGF. 20–30 kcal/kg/d for patients in remission, 10–20% more for malnourished patients or those with an active disease. Foods were strictly forbidden except for water. Anti-inflammatory agents not allowed in the 12 weeks patients were on this diet	Induction of Remission in active CD	N/A	N/A	12 weeks of EN therapy improvements in nutritional state and remission. Patients with CDAI of <150 were considered to be in clinical remission. BMI, albumin, hemoglobin, and inflammatory index (high sensitivity C-reactive protein, platelet, and erythrocyte sedimentation rate (ESR)), were used to determine nutritional status.	N	43/44	In patients with stenosis, 6/10 had complete remission, 2/10 had partial remission, and 2/10 had no response to EEN and were transferred for surgery. hs-CRP: 62% decrease Albumin: 18% increase	NS	In patients with stenosis, 1 patient could not tolerate EEN at week 2 due to diarrhea and lack of change in symptoms.
Teahon, 1990	UK	R	No definition provided	Chart review, history of DI (did not specify type of DI).	113	35	ED. Vivonex. Nitrogen was given depending on the extent of nitrogen depletion and energy. Starter regimens were used beginning the feed at 1/3 strength and 1800 ml. 3-day period the osmolality was increased to full strength (550 mmol/L) and volume is increased. Ninety-six took the diet of and 17 required NGF. Patients were on diet for an average of 4 weeks.	Treatment of CD	Malnutrition	60 months	Immediate and long-term outcome of treating patients with CD with an elemental diet, followed by diet remission. (Defined by need to change therapy or failure to achieve remission as per physician, no scores used)	N	106/113	33/35 symptomatic strictures achieved remission. 13/21 remission on normal food (mean 22 months, range 1–108 months) CRP: NS Albumin: NS	NS	NS
Ostro, 1985	Canada	R	Obstruction	Chart review and DI (modality not specified).	100	29	TPN in 3 groups. Group 1: (*n* = 75) 50% glucose, 50% lipid (Intralipid). Group 2: (*n* = 15) 83% lipid formulation (Intralipid) and 1 g/kg ideal body weight per day of protein (either Amigen or Travasol) and 40 kcal/kg ideal body weight per day of nonprotein calories. Group 3: (*n* = 10) mean protein input of 1.7 g/kg/day in the absence of additional nonprotein caloric supplementation. TPN provided for 25 days on average.	Treatment of CD	N/A	3 and 12 months	Clinical remission defined as the absence of obstructive symptoms once oral food was re-introduced, or a decreased CDAI score < 150.	N	NS	Clinical remission occurred in 22/29 patients with subacute bowel obstruction. CRP: NS Albumin: NS	NS	NS
Marafini, 2020	Italy	R	Presence of localized thickening of the intestinal wall associated with lumen narrowing, with or without proximal bowel dilation.	File review, record of at least one stricture found using CEUS or MRE.	82	82 (37 on diet)	Liquid Diet. Consumed only beverages (e.g., water, tea, juices) for 24 h every 10–14 days.	Improve management of stricturing CD	N/A	12 months	Trial using 1 day of liquids-only diet every 10–14 days to reduce surgical interventions due to intestinal strictures.	N	NS	10/37 of the stricture patients in the liquid diet group had sub-occlusive episodes and 11/37 had surgical resections due to sub-occlusive episodes being unresponsive to therapy. CRP: NS Albumin: NS	NS	NS

BMI, Body mass index, CD, Crohn’s disease; CDAI, Crohn’s disease activity index; CEUS, contrast enhanced ultrasound; CR, Clinical remission; CRP, C-Reactive Protein; CT, computed tomography; DI, diagnostic imaging; EEN, exclusive enteral nutrition; EN, elemental nutrition; hs-CRP, high sensitivity C-Reactive Protein; IBD, inflammatory bowel disease; MRE, magnetic resonance enterography; NGF, nasogastric feeding; NS, not stated; RR, Radiologic remission; SR, Symptomatic remission; TPN, total parenteral nutrition.

### Risk of bias and quality assessment in individual studies

The quality of non-randomized studies was assessed by using the Newcastle-Ottawa Scale (15). The quality of the studies was examined for: (1) Selection, (2) Comparability, (3) Outcome (Supplementary Table S1). The Grading of Recommendations Assessment, Development and Evaluation (GRADE) system was then used to determine the quality of the evidence as high, moderate, low, or very low (Supplementary Table S2).

## Results

### Search and screening results

**Figure 1:**
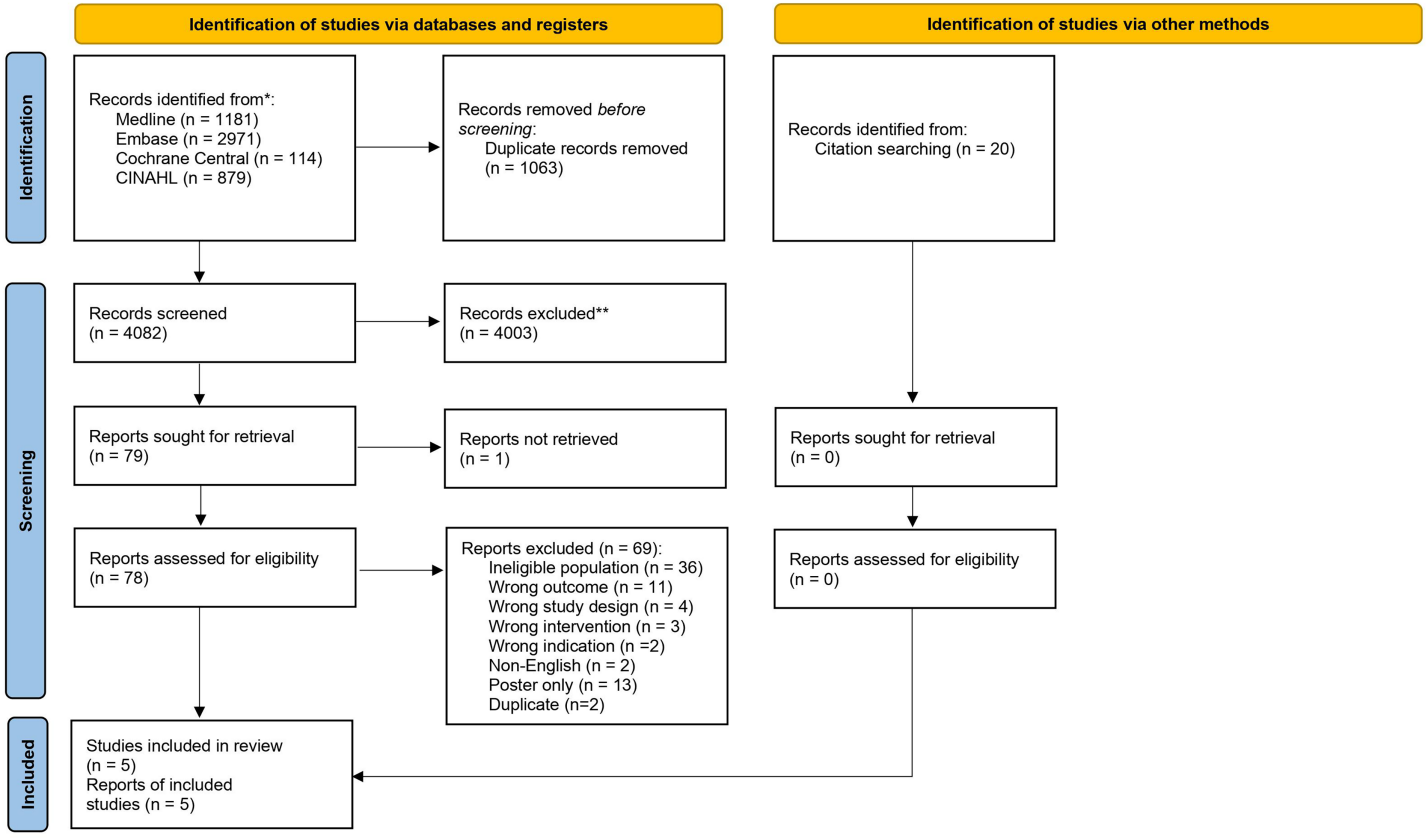
PRISMA flow diagram displaying databases searched, number of articles screened, and overall study selection.

A total of 5,145 records were identified in the initial search, of which there were 1,063 duplicates. 4,082 records were screened by title and abstract for eligibility. From these, 4,003 records were removed due to irrelevance. A total of 79 records underwent full text review and a total of five studies were identified as eligible for inclusion in this review (Figure 1). Of the studies included in the review, three were retrospective cohort studies, two were prospective cohort studies, and no studies were randomized controlled trials. There were three studies which evaluated exclusive enteral nutrition (EEN), one study which looked at total parenteral nutrition, and one study of a liquid diet.

### Exclusive enteral nutrition

There were two prospective cohort studies (20, 21), and one retrospective study (22), that investigated the role of exclusive enteral nutrition (EEN) in fibrostenotic CD.

In the first prospective study assessing EEN, Hu et al. recruited 65 patients with fibrostenotic CD to complete 12 weeks of treatment (20). EEN was provided *via* nasogastric or nasogastrointestinal tube for 12 weeks using 1,500 to 2,000 mL (1calorie/mL per day) of elemental formula (Peptisorb Liquid), with energy requirements calculated by Long’s equation modified by usual body weight (23). Oral food or fluid was not permitted during the study period except for water and tea. Compliance with EEN was defined as intake of >80% of the prescribed treatment dose. Patients who were treated with corticosteroids, immunosuppressants, or infliximab within 3 months of the study start date were excluded. Aminosalicylates, rectal steroids, azathioprine, 6-mercaptopurine, cyclosporine, other immune-suppressants, and parenteral nutrition were also not permitted during the 12-week study period.

Stricture diagnosis was determined by medical history, clinical presentation, physical examination, CT imaging, and endoscopy. Clinical symptoms used to diagnose stricture were abdominal pain, abdominal distention, vomiting, and constipation. The most common presenting symptoms of the included 59 patients was abdominal pain (59.3%), abdominal distention (55.9%), and constipation (39.0%). Physical examination findings utilized was abdominal tenderness and tension, and hyperactive bowel sounds. Blinded review of patient CT scans was conducted to evaluate BWT, bowel loop diameter of dilation, and luminal diameter of stricture. However, specific diagnostic criteria and cut-offs for small bowel stricture features such as BWT, luminal apposition and prestenotic dilation on abdominal CT were not specified. Strictures were further stratified into inflammatory or fibrotic using elevated laboratory variables (C-reactive protein (CRP), erythrocyte sedimentation rate (ESR), white blood cell (WBC) count, and lymphocyte count), and CT findings of “string sign” and thickened bowel wall. Patients with a fibrous subtype of stricture, classified as an absence of inflammatory laboratory markers, were excluded from the study.

There were 65 patients recruited, with 59 studied because of dropout (two) and poor compliance (four). Of the 65 patients studied, 50 completed the 12 weeks of therapy and nine patients required surgery during the course of the study. Of these 50 patients who completed EEN, all had fibrostenotic behavior (B2) according to the Montreal classification of CD. The remaining nine study participants had inflammatory behavior (B1). Neither the location of the strictures (ileal vs. colonic), or presence of multiple of strictures was reported. The disease location in the patients studied was 40.7% (24/59) ileal (L1), 42.4% (25/59) ileocolonic (L3), and 16.9% (10/59) colonic (L2). In addition, 6.8% (4/59) had upper gastrointestinal disease (L4), and perianal disease was present in 8.5% (5/59). The number of patients with previous bowel resection was also not described, however the authors do state that patients with <200 cm of bowel remaining post resection were excluded.

Clinical remission was defined as a decrease in CDAI score decrease of >70, and the primary outcome was the proportion of patients to achieve this by week 12 (20). Secondary endpoints included subjective symptomatic improvement, radiographic remission defined as 100% increase in luminal cross-sectional area on CT, and complete remission was deemed if patients met all primary and secondary endpoints. Of the patients included in analysis, 71.2% (42/59) patients achieved clinical remission at 12 weeks. Of the 50 participants who completed the 12 weeks of EEN, there was a significant reduction in CDAI scores from baseline of 188.2 ± 17.46 to 132.4 ± 10.27 (*p* < 0.05) at 4 weeks and 92.9 ± 10.95 (*p* < 0.05) at 12 weeks. There was evidence of a statistically significant decrease in CRP (28.18 ± 4.84 mg/L to 10.44 ± 2.30 mg/mL, *p* < 0.05), increase in body mass index (BMI) (19.19 ± 0.29 Kg/m^2^ to 20.78 ± 0.22 Kg/m^2^, *p* < 0.05) and albumin (31.36 ± 0.66 g/L to 40.43 ± 0.73 g/L, *p* < 0.05) at 12 weeks.

Regarding imaging findings, CT imaging showed a decrease in BWT at 12 weeks (8.68 ± 0.37 mm to 3.53 ± 0.04 mm, *p* < 0.05) and an increase in luminal cross-sectional area (59.09 ± 10.64 mm^2^ to 195.7 ± 18.79 mm^2^, *p* < 0.05). Symptomatic remission was achieved by 81.4% (48/59) of patients and 59.3% (35/59) of patients achieved radiographic remission. Overall, 50.8% (30/59) of patients achieved complete remission while 15.3% (9/59) of patients had progression in obstructive symptoms requiring surgical resection during the first 4 weeks of study involvement. Of these nine patients requiring surgery, four had confirmed fibrotic strictures post-surgery. The remaining five patients were considered to have inflammatory strictures, however histologic criteria of a stricture were not specified. Four of the nine patients received small bowel resection with anastomosis, and five received ileocecal resection with ileocolonic anastomosis. No patients were reported to have received EBD intervention.

The second prospective study evaluating EEN included in this review was by Yang et al. who prospectively followed 41 adult patients with CD and associated complications defined as enteric fistula, perienteric abscess, or stricture. More specifically, 10 patients had fibrostenotic behavior treated with EEN over 12 weeks (21). EEN was administered though nasogastric tube with a daily energy supplementation of 20–30 kcal/kg/d for those in remission, and 10 to 20% more for those with either malnourishment and/or active disease. Malnourishment was evaluated using the European Nutritional Risk Screening 2002 tool at baseline and after 12 weeks of EEN. All oral food intake was strictly forbidden, and water was permissible. Patients were not permitted treatment with any steroids, biologics, or immunomodulators over the 12-week study period. Baseline use of these medications prior to study period was not reported. Presence of obstructive symptoms in patients was not assessed. Although stricture diagnosis was confirmed by CT, CT enterography (CTE), magnetic resonance enterography (MRE) and endoscopy, specific diagnostic imaging criteria of stricture were not detailed. Patients with strictures underwent further evaluation using intravenous contrast enhanced intestinal ultrasound to distinguish between inflammatory and fibrotic components as assessed by the percentage of maximal wall enhancement. A size cut-off for BWT, stricture length, luminal diameter, bowel loop diameter, and prestenotic bowel dilation was not reported.

Of the initial 44 patients recruited, 41 were evaluated (one dropout, two lost to follow-up). Of these 41 patients who completed the therapy, eight were noted as having stricturing behavior (B2) with no specified stricture location, and the remaining were classified as having penetrating behavior (B3). The authors note that there was a total of 10 stenotic patients where two patients had concurrent fistulizing disease. All enrolled patients were classified as either ileocolonic with or without upper disease (Montreal Classification L3 or L3 + L4). Twelve patients were reported to have had previous intestinal surgery; however, the number of surgeries due to stricturing and/or fistulizing CD were not specified.

The primary outcome were rates of clinical remission and mucosal healing measured at baseline, week 4, and week 12. Clinical remission was defined as a CDAI score of <150, and mucosal healing was defined as a simple endoscopic score for Crohn’s disease (SES-CD) of <2. Rates of partial remission were reported, however no definition was provided. Secondary outcomes were nutritional markers including BMI, albumin, hemoglobin, and laboratory inflammatory measures of high sensitivity CRP, platelets, and ESR at baseline, week 4 and week 12. CTE or MRE was conducted at baseline and week 12 to evaluate penetrating and stricturing CD characteristics, however the metrics collected for strictures was not detailed. Of the 6/10 (60%) of patients with stricture, 6/10 (60%) achieved clinical remission, 2/10 (20%) of patients achieved partial remission, and 2/10 (20%) patients failed to achieve remission requiring surgery. Twenty-three patients underwent paired colonoscopy or double balloon enteroscopy at baseline and week 12, however it is not indicated how many of the stricturing patients received endoscopic balloon dilation (EBD). One patient with stenosis could not tolerate EEN due to diarrhea and lack of improvement in symptoms at week 2. The alternate treatment received for this patient was not reported. Two patients were reported to have relapsed with obstructive symptoms and were transferred to surgery at week 10 and week 12. All 10 stricturing CD patients failed to achieve a statistically significant decrease in bowel thickness with a BWT of 9.07 ± 4.01 mm at baseline compared to 7.48 ± 2.97 mm (*p* = 0.34) after 12 weeks of EEN.

In the only retrospective study assessing EEN in fibrostenotic CD, Teahon et al. analyzed 113 hospitalized CD patients, 35 of whom had symptomatic strictures, treated with either elemental diet alone or elemental diet in addition to steroids for flare symptoms (22). These patients receiving an elemental diet were compared to 37 patients treated with steroids only. Elemental diet was given as the sole source of nutrients for an average of 4 weeks (range 2–12). For patients who were unable to tolerate the poor palatability of the diet, despite cooling, flavoring, or drinking through a straw, administration was conducted with a fine bore nasogastric tube. The elemental formula used was Vivonex, with nitrogen provided at 0.17–0.30 g/kg/day depending on the extent of depletion, with overall energy between 8.4–12.6 MJ (approximately 2,000–3,000 kcal). It was not stated if any concomitant medications apart from steroids were used in those received the diet, as the authors state the elemental diet was given alone or as part of treatment programme. Patients receiving steroids (prednisolone) were given a dose of 0.75 mg/kg/day which was then gradually reduced, however the period for taper was not reported. Stricture definition and specific imaging parameters for strictures was not provided by the authors, but diagnosis was reported to be made from chart review and history of diagnostic imaging (modality not specified). Obstructive symptoms were not reported or defined; however, patients are noted as having symptomatic strictures. Of the 35 stricturing patients, nine were classified has having disease located in the small bowel; 15 with terminal ileal distribution, and 11 with ileocolonic. Past surgical history in patients with strictures was not reported.

Immediate and long term (60 months) remission after elemental diet were assessed in this study. Remission was determined by both the patient and physician, however the specific criteria assessed were not described. Failure of treatment was noted as a lack of improvement at the end of the treatment, or if the treatment plan required alteration due to deterioration or intolerance to therapy. Objective markers with diagnostic imaging and/or laboratory inflammatory markers pre and post EEN were not reported. Thirty-five of these patients had confirmed strictures of which 33 patients reported symptomatic remission. Twelve (12/35; 34.2%) patients went for elective surgery at the end of their dietary treatment, twenty-one (60%) patients returned to a normal diet, of which 13 (37.1%) maintained remission (range 1–108 months) and 8 had relapsed (range 1–96 months). The number of stricture patients who received diet alone versus diet and corticosteroid was not reported. No patients were reported to have received EBD intervention.

### Total parenteral nutrition

One retrospective study assessed the use of total parenteral nutrition (TPN) in the management of CD (24). Ostro et al. looked at the records of 100 hospitalized CD patients treated with TPN, of which 29 were classified as having a subacute obstruction (24). The diagnostic criteria for a subacute obstruction were not described. Mean TPN treatment duration was 25.5 ± 1.1 days with a range of 7 to 76 days. Three systems of TPN were provided in this study (Table 1). The first system was a 50% glucose 50% lipid combination, and the second was an 83% lipid formulation, where a non-protein caloric target of 40 kcal/kg of ideal body weight was used. Protein was provided at 1 g/kg of ideal body weight as casein hydrolysate or as a defined amino acid mixture. The third system used a protein input of 1.7 g/kg/day. All enrolled patients were reported to have had at least one unsuccessful prior treatment with steroids either alone, or in addition to sulfasalazine or azathioprine. Use of sulfasalazine, azathioprine, antidiarrheal agents, and analgesics were discontinued prior to beginning TPN, however steroid use was not. A definition for stricture was not reported in this study, however the stratification of patients was performed based on their clinical, radiographic, and histologic data. Characteristic stricture diagnostic imaging parameters and surgical history was not reported. The location of disease in patients with subacute bowel obstruction was 58.6% (17/29) small bowel, 3.5% (1/29) large bowel, and 37.9% (11/29) both small and large bowel.

The primary endpoint of this study was clinical remission defined as an absence of recurrent obstructive symptoms once oral intake was re-introduced, or a decrease in modified CDAI score to <150. Relapse in the follow-up period of 3 months and 1 year was defined as either the commencement, or dose increase of steroids, or readmission to the hospital. Overall, clinical remission was obtained in 75.8% (22/29) of patients at 3 months. There was a statistically significant drop of the average modified CDAI score from approximately 300 to <150, *p* < 0.001. At 3 months follow up, one patient was lost to follow-up, and 78.6% (22/28) patients with subacute bowel obstruction remained in remission. At 1 year, 57.7% (15/26) were in remission, and two additional patients were lost to follow-up. During the treatment period, 17.2% (5/29) of patients with subacute obstruction required surgery. In addition, one patient who presented with an inflammatory mass developed a bowel obstruction and required surgery. At 3 months follow up, 3.6% (1/28) required surgery with no relapses, and at 1 year 11.5% (3/26) required surgery. No patients were reported to have received EBD.

### Liquid diet

One retrospective case control study assessed the use of a liquid diet in stricturing CD (25). Marafina et al. assessed the administration of a liquid diet (only water, tea, juices) without solid food for a 24-h window every 10 to 14 days, in addition to their conventional treatment (anti-tumor necrosis factor (TNF), vedolizumab, thiopurines, steroids, mesalamine, or antibiotics). The 10 to 14-day period was selected based on perceived patient tolerability. Thirty-seven small bowel stricture patients who undertook this diet were compared to 45 patients with strictures without modified diet. The follow-up period for this study was 12 months, and strictures were defined as the presence of a localized thickening of the intestinal wall associated with luminal narrowing, with or without proximal bowel dilation on either oral contrast enhanced ultrasound or MRE. Specific cut-off values for criteria for BWT, prestenotic dilation, and luminal diameter was not reported. The primary outcomes of this study were the occurrence of sub-occlusive episodes and subsequent requirement for bowel resection following a liquid diet, compared to standard treatment after 12 months. However, the authors did not define a sub-occlusive episode.

The primary outcome of sub-occlusive episodes was evaluated in three distinct groups: (1) proximal bowel dilation vs. those without dilation, (2) previous resection vs. those without resection, and (3) concomitant steroids vs. those not on steroids. At the 12 month follow up period, there was no statistically significant differences in sub-occlusive episodes within each group, with 27% (10/37) in the diet group reporting occurrence, as compared to 20% (9/45) in the control group (*p* = 0.45). Similarly, the rate of bowel resection due to obstruction did not differ between groups, with 24.3% (9/37) in the diet group and 15.5% (7/45) in the controls (*p* = 0.07) requiring surgery. The presence of prestenotic dilation, use of corticosteroids, or having a previous resection did not influence the results. No patients were reported to have received EBD intervention.

All strictures were located in the small bowel and 32.4% (12/37) of the intervention group had a previous resection. Average stricture length at baseline was similar between groups and was reported as median of 20 cm (range 10–30 cm) in the group receiving liquid diet, and 15 cm (8–20 cm) in those who received no intervention (*p* = 0.14). 56.8% (21/37) of patients who received the liquid diet had proximal bowel dilation at baseline, with significantly less bowel dilation 20% (9/45) in the no intervention group (*p* = 0.001). Interestingly, no patients had obstructive symptoms related to their stricture at baseline. Patients in the diet group had significantly more steroid therapy [46.7% (21/45), *p* = 0.04], when compared to the control group [24.3% (9/37)]. There was no statistical difference between groups for any of the other medical treatments used.

## Discussion

Diet and nutrition play an important role in the management of Crohn’s disease and is of substantial interest to many patients who want a dietary therapeutic option (26). Evidence is accumulating for the role of diet and nutrition for CD induction and maintenance of remission, and impact on natural history (10, 15, 27, 28). Levine et al. evaluated the Crohn’s disease exclusion diet (CDED) coupled with partial EN, versus EEN alone in the first randomized controlled trial of its kind in children with mild to moderate CD over 12 weeks (8). The CDED plus partial EN was better tolerated than EEN and induced clinical and biomarker remission. In terms of the stricturing phenotype, it is presumed that patients with strictures were excluded as only those with mild to moderate luminal disease were included (8). Nonetheless, while research into diet therapies for CD induction and maintenance of remission is gaining momentum, their efficacy in adult CD remains poorly understood and specifically, the impact upon fibrostenotic phenotype is inadequately defined in most studies. Furthermore, most studies utilize symptom indices such as the CDAI as a measurement of stricture improvement post dietary intervention. However, it is well known that symptoms do not necessarily correspond with extent and severity of CD. This systematic review summarizes the available evidence for using dietary interventions in the management of fibrostenotic CD.

EEN is the most studied form of dietary therapy in fibrostenotic CD. Our search identified two prospective cohort studies (20, 21), and one retrospective study (22), evaluating the use of EEN in adult CD with strictures. Both prospective EEN studies had similar results, achieving clinical remission in approximately 60% of fibrostenotic CD after 12 weeks of EEN treatment (20, 21). Although these are positive findings, limitations include a lack of well-described definitions for strictures, a short duration of study, and lack of long-term outcomes. As well, the use of CDAI, where scoring is largely affected by the frequency of bowel movements may provide a limited clinical assessment of disease severity in patients with fibrostenotic CD, who may not have such high frequency. There are currently no validated scoring systems of patient reported outcomes (PRO) specific to this population (7). The single retrospective study assessing EEN as an intervention found similar efficacious results with 33/35 stricture patients achieving clinical remission as defined by a return to pre-relapse well-being (22). More specifically, patients on EEN alone were compared to those treated with corticosteroids and EEN, as well as those with corticosteroids only. There was no significant difference between these groups for the primary outcome of achieving clinical remission, however the number of patients with strictures in each study group was not described. In addition, it is unclear how the daily energy requirements for EEN dosing were calculated for each patient. Other limitations include a lack of objective measures of stricture improvement, and although laboratory markers such as CRP, ESR, and albumin were measured, changes in these measures were not specifically reported for the stricture group. At this time, literature on the use of EEN in adult CD has reported widely varying rates of clinical remission, defined as a decrease in CDAI, or Harvey Bradshaw Index (HBI) with a range of 27–100% achievement in those that completed the therapy (29). Recorded improvements include mucosal and transmural healing (30, 31), decreased inflammatory markers (31, 32), and symptomatic remission (32). However, limitations of the majority of studies include an absence of clearly defined phenotypes. Overall, there is limited data evaluating EEN in adult CD and particularly for the stricturing subtype.

EEN has been primarily utilized as a successful therapy in pediatric CD populations in lieu of corticosteroids (33). It is thought that perhaps the palatability of EEN supplements and tolerability of tube-feeds is a limitation in adults, but recently a retrospective study in the UK compared 2 weeks of pre-operative oral enteral nutrition (EN) in 173 CD patients versus no nutritional optimization in 96 patients (34). It was found that patients undergoing surgery with pre-operative EN had improved post-operative outcomes as measured by significantly lower 30 day post operative complications, reductions in pre-operative CRP and improved albumin (34). A short duration of EEN such as 2 weeks appears to be well tolerated in adults and can still provide benefit. Furthermore, the use of EEN pre-surgery in patients with stricturing or fistulizing CD complications also demonstrated benefit, where 25% of patients improved substantially and no longer required surgery. In those who received surgery, better outcomes in comparison to controls were noted (35).

Although a documented drawback of EEN is poor compliance rates due to palatability, a high rate of adherence was identified in the prospective studies analyzed in this systematic review (20, 21, 29). In these studies over a 12-week period, only 9/109 dropped out due to non-compliance, lost to follow up (21), or other reasons (20, 21). Administration of EEN during this period was either through nasogastric tube (21), or multiple oral suspensions during the daytime, with a self-intubated nasogastric tube at night (20). Similar previous studies have also reported higher tolerance to EN with a nasogastric route (36). Controlled trials investigating long-term (>12-week) use of EEN in adults are needed to appropriately assess compliance. An additional limitation in all included EEN studies was heterogeneous definitions for stricturing CD. The study by Hu et al. defined strictures *via* medical history, clinical presentation, and physical examination (20). This retrospective study did not state how they determined if patients were of the stricturing subtype (22). Additional diagnostic criteria from cross-sectional imaging such as prestenotic dilation, wall thickening, and luminal narrowing, could aid in clear stricture classification for future studies (7, 37). Furthermore, in the two prospective studies included in this review, both sought to exclude fibrotic strictures and include only inflammatory strictures and utilized cross-sectional imaging (enhanced ultrasound or CT) to distinguish between. The rationale for this methodology was due to the likely mechanism of action for EEN, which has been well studied and shown to be effective with inflammation (33). However, active and chronic strictures are known to contain components of both inflammation and fibrosis (38). More specifically, in the study by Hu et al. 4/9 patients requiring surgery had confirmed fibrotic strictures (20). This suggests that the use of EEN may not be as efficacious in a primarily fibrotic stricture, as compared to an inflammatory stricture as inflammation is thought to respond best to medical therapy, including EEN (39). This is further supported by the results from Yang et al. where they attributed the non-significant reduction in bowel wall thickness to strictures of a mixed inflammatory and fibrotic components, rather than predominantly inflammation alone. Another limitation of these studies is the lack of comparison to those with an inflammatory CD phenotype, as well as differentiation of an anastomotic stricture vs. a *de novo* stricture, as these may behave differently to therapy. In addition, a limitation in these studies is the absence of a control group for comparison which does limit the degree to which EEN can be promoted in stricturing CD.

EEN as an intervention in CD is often delivered for less than 1 year. However, in one study evaluating anti-TNF therapy with concomitant elemental diet supplementation over 2 years, it was found that relative to the control group without the diet, there was no change in remission rates after baseline (40). When analyzing predictors for disease relapse, the authors found a hazard ratio of 3.64 (1.58–8.39) for intestinal strictures (40). In a retrospective study investigating enteral nutrition and hospitalization rates in 268 CD patients over 5 years, researchers found that those with isolated ileal disease compared to those without this disease distribution had lower hospitalization rates when supplemented with EN of ≥900 kcal/day (41). Multivariate analysis revealed that those with strictures had a hazard ratio of 2.31 (1.57–3.33) for hospitalization despite EN therapy (41). This study had limited details of stricture location, definition, and concurrent medications to draw other conclusions. Overall, limitations of all studies included in this systematic review are poor stricture characterization, as well as a lack of in-depth details of concurrent or past biologic therapy, making conclusions, recommendations about diet therapy, and applicability to real-life practice challenging.

EEN is a major focus of most nutrition related studies and only one paper investigating TPN specifically in fibrostenotic CD was found in our search. In the study by Ostro et al. from 1985, of the 29 patients classified as having obstruction 22 achieved clinical remission at 3-month follow up, defined as a CDAI score of <150 (24). This study did not describe if the patients with subacute bowel obstruction had received diagnostic imaging to delineate the presence of a stricture, and the definitions utilized as classification was completed by chart review. In addition, it cannot be determined from the patient group who presented with bowel obstruction if there was concurrent use of corticosteroids throughout the study period, and the specific details of TPN therapy provided was unclear. An additional limitation was the lack of a control group for comparison. In an additional retrospective cohort study not included in our systematic review due to a lack of the number of stricture patients specified, post-surgical outcomes of patients receiving TPN prior to resection were analyzed (42). The results from this study were similar to those found by Ostro et al. as they noted that all 20 patients included demonstrated significant improvements in clinical, laboratory, and radiographic assessments (42). However, results in this study were pooled to contain both stricturing and penetrating CD, making precise conclusions about the benefit of TPN in strictures challenging. Further research evaluating the use of TPN in patients with strictures with well-defined outcomes is needed to evaluate the efficacy of this diet in fibrostenotic CD.

Studies investigating alternative diets were limited in our literature search, and only one study evaluating a liquid diet was found. This retrospective case–control study found no difference in the occurrence of new obstructive episodes or the rate of bowel resection between controls and those that underwent a liquid diet (water, tea, juice, etc.) for a 24-h period once every 2-weeks (25). Stricturing was evaluated using cross-sectional imaging (MRE or CEUS) and was defined as having localized bowel wall thickening and luminal narrowing, with or without prestenotic dilation. It is worth noting that there was a significant difference at baseline between the two groups in both steroid use (higher in controls) and presence of prestenotic dilation (higher in intervention) (25). Additionally, another study investigated the use of a plant-based diet in biologic naïve CD patients undergoing standard induction therapy with infliximab, and reported significant reductions in CDAI and CRP after 6 weeks (43). However, this population included both adults and children of mixed CD phenotype and did not include individual analysis of the stricturing subtype, or a control group for comparison. This diet is likely not applicable to patients with fibrostenosis as current expert recommendations for dietary treatment of fibrostenotic CD is low amounts of insoluble fiber (44). Perhaps a plant-based diet rich in soluble fiber and resistant starch, while limiting insoluble fiber is a potential option in strictures requiring further study.

To our knowledge, our study is the first systematic review to evaluate rates of surgical resection and clinical remission post nutritional therapy in established small bowel CD strictures. However, there are limitations primarily due to the quantity of data and limited definitions of strictures on diagnostic imaging that were also not confirmed histologically in those who underwent a resection. The main weakness is the lack of consistency of therapy, duration, and the missing details of stricture severity, definitions, and whether the stricture is anastomotic or naive. Many studies utilized CDAI or HBI to measure clinical remission as a primary outcome. It is well known that although symptoms are an important measure, well-controlled markers such as fecal calprotectin and diagnostic imaging parameter improvements are associated with decreased hospitalization and better disease outcomes (45). Comparisons across studies were challenging with follow up length, concurrent medication use, and strictures characteristics. As no randomized controlled trials were available, a meta-analysis could not be performed and caution in data interpretation is required. Another limitation of this systematic review includes the exclusion of pediatric patients. It is unclear if data from the pediatric population could be extrapolated to adults. Nonetheless, there are important conclusions that can be drawn and another future review to include the pediatric population would be warranted. In addition, despite low-fiber diets being a well-known recommendation in clinical practice, no papers discussing fiber modification, low residue diets, or low fermentable oligosaccharides, disaccharides, monosaccharides, and polyols (FODMAP) as an intervention for fibrostenosis were identified during our search. Research using these dietary modifications for patients with fibrostenotic CD is therefore also warranted.

In conclusion, this systematic review has found that EEN or TPN may provide a benefit in symptom reduction in fibrostenotic CD, however there are too few studies with heterogeneous stricture criteria to formulate recommendations at this time. Despite the fact that a great proportion of CD patients with strictures lack symptoms, the majority of current available studies are dependent on symptomatic outcomes (46). As a result, there is a need for objective measures of strictures using cross-sectional imaging modalities to evaluate hallmark stricture features such as bowel wall thickness, luminal apposition, pre-stenotic dilation and overall transmural healing in relation to nutritional interventions (46). Further research on diet and nutrition in fibrostenotic CD is needed to determine efficacy of interventions such as exclusion diets and enteral nutrition in management during both periods of flare and remission, along with concurrent medical therapy which is so often excluded in studies. Additionally, as many patients with strictures will require bowel resections to manage their disease, diet and nutrition has an important role in both the pre and post operative period that also requires further evaluation. As there is a strong interplay between nutrition and CD activity and phenotype, the impact of nutrition and current medications coupled with future anti-fibrotic therapies will be of great interest.

## Data availability statement

The original contributions presented in the study are included in the article/Supplementary material, further inquiries can be directed to the corresponding author.

## Author contributions

CL and MR contributed to the conception or design of the work. ZP conducted the database searches. JC and AF reviewed inclusion and exclusion of abstracts and manuscripts. JC, RR, and CL contributed to acquisition and interpretation of data. JC, RR, MR, and CL drafted and edited the manuscript. JC, RR, AF, ZP, HS, RI, GK, CM, KN, RP, CS, FR, MR, and CL have read, edited, and approved the final manuscript.

## Conflict of interest

The authors declare that the research was conducted in the absence of any commercial or financial relationships that could be construed as a potential conflict of interest.

## Publisher’s note

All claims expressed in this article are solely those of the authors and do not necessarily represent those of their affiliated organizations, or those of the publisher, the editors and the reviewers. Any product that may be evaluated in this article, or claim that may be made by its manufacturer, is not guaranteed or endorsed by the publisher.

## Abbreviations

BMI: Body Mass Index
CD: Crohn’s Disease
CEUS: Contrast Enhanced Ultrasound
CDAI: Crohn’s Disease Activity Index
CRP: C-Reactive Protein
CTE: Computed Tomography Enterography
EBD: Endoscopic Balloon Dilation
EET: Exclusive Enteral Nutrition
EN: Enteral Nutrition
FODMAP: Fermentable Oligosaccharides, Disaccharides, Monosaccharides, and Polyols
IBD: Inflammatory Bowel Disease
MRE: Magnetic Resonance Enterography
NSAID: Non-Steroidal Anti-Inflammatory Drug
SES-CD: Simple Endoscopic Score – Crohn’s Disease
TNF: Tumor Necrosis Factor
TPN: Total Parenteral Nutrition
