# Supplementary material for: Nutritional interventions in adult fibrostenotic Crohn’s disease: A systematic review
Source: Front Nutr. 2023 Feb 21;10:1017382. doi: 10.3389/fnut.2023.1017382 (PMC9988909; doi:10.3389/fnut.2023.1017382)
Supplement: Supplementary file 1 [file Table_1.DOCX]

**Table S3: Search strategies for Medline (Ovid)**

Database: Ovid MEDLINE(R) and Epub Ahead of Print, In-Process, In-Data-Review & Other Non-Indexed Citations and Daily <1946 to March 27, 2022>

| **#** | **Query** | **Results from 27 Mar 2022** |
| --- | --- | --- |
| 1 | inflammatory bowel diseases/ or crohn disease/ or ileitis/ | 66,764 |
| 2 | Crohn*.tw,kf. | 52,937 |
| 3 | ("Inflammatory bowel disease*" or IBD).tw,kf. | 62,400 |
| 4 | ((regional* or terminal) adj3 (ileiti* or enteritis or enterocolitis)).tw,kf. | 2,176 |
| 5 | 1 or 2 or 3 or 4 | 103,901 |
| 6 | Constriction, Pathologic/ | 32,686 |
| 7 | Intestinal Obstruction/ | 30,811 |
| 8 | Fibrosis/ | 35,852 |
| 9 | ((gut or colon or colonic or ileal or ileum or intestin* or duodenal or duodenum or jujenal or jujenum or abdominal or "intra-abdominal" or anastomotic) adj5 (strictur* or constriction* or stenos* or atresia* or obstruct* or narrowing or lesion* or fibros* or abscess* or lesion* or "wall thick*" or perfusion* or dilation)).tw,kf. | 66,109 |
| 10 | (bowel adj5 (strictur* or constriction* or stenos* or atresia* or obstruct* or narrowing or lesion* or fibros* or abscess* or lesion* or "wall thick*" or perfusion* or dilation)).tw,kf. | 18,575 |
| 11 | (strictur* adj3 (lesion* or fibrotic or inflammatory or complication* or disease)).tw,kf. | 3,188 |
| 12 | 6 or 7 or 8 or 9 or 10 or 11 | 157,737 |
| 13 | 5 and 12 | 7,102 |
| 14 | Crohn Disease/dh [Diet Therapy] | 499 |
| 15 | Inflammatory Bowel Diseases/dh [Diet Therapy] | 341 |
| 16 | Ileitis/dh [Diet Therapy] | 10 |
| 17 | exp Diet/ | 312,099 |
| 18 | enteral nutrition/ or nutrition therapy/ or exp diet therapy/ or nutritional support/ or exp Parenteral Nutrition/ | 109,838 |
| 19 | exp Food/ | 1,392,668 |
| 20 | (diet? or dietary or nutrition* or nutrient* or food? or feed or feeding).tw,kf. | 1,457,579 |
| 21 | (fasts or fasting or weight or obes* or BMI or body mass index or quetelet or body fat).tw,kf. | 1,367,617 |
| 22 | body mass index/ | 142,068 |
| 23 | exp body weight/ | 503,887 |
| 24 | (supplement? or vitamin? or mineral? or carbohydrate? or lipid?).tw,kf. | 1,101,402 |
| 25 | microbiota/ or gastrointestinal microbiome/ | 55,041 |
| 26 | ((gut or gastr* or intestin*) adj3 (microbio* or microflora or flora)).tw,kf. | 58,848 |
| 27 | 14 or 15 or 16 or 17 or 18 or 19 or 20 or 21 or 22 or 23 or 24 or 25 or 26 | 4,264,144 |
| 28 | 13 and 27 | 1,181 |

**Table S4: Search strategies for Embase (Ovid)**

Database: Embase 1974 to 2022 March 27

| **#** | **Searches** | **Results** |
| --- | --- | --- |
| 1 | *inflammatory bowel disease/ or *crohn disease/ or *ileitis/ | 75649 |
| 2 | Crohn*.tw,kf. | 92173 |
| 3 | ("Inflammatory bowel disease*" or IBD).tw,kf. | 110961 |
| 4 | ((regional* or terminal) adj3 (ileiti* or enteritis or enterocolitis)).tw,kf. | 1094 |
| 5 | 1 or 2 or 3 or 4 | 163223 |
| 6 | Constriction, Pathologic/ | 3605 |
| 7 | small intestine obstruction/ or intestinal fibrosis/ or intestine stenosis/ | 15224 |
| 8 | Fibrosis/ | 84259 |
| 9 | ((gut or colon or colonic or ileal or ileum or intestin* or duodenal or duodenum or jujenal or jujenum or abdominal or "intra-abdominal" or anastomotic) adj5 (strictur* or constriction* or stenos* or atresia* or obstruct* or narrowing or lesion* or fibros* or abscess* or lesion* or "wall thick*" or perfusion* or dilation)).tw,kf. | 87110 |
| 10 | (bowel adj5 (strictur* or constriction* or stenos* or atresia* or obstruct* or narrowing or lesion* or fibros* or abscess* or lesion* or "wall thick*" or perfusion* or dilation)).tw,kf. | 28638 |
| 11 | (strictur* adj3 (lesion* or fibrotic or inflammatory or complication* or disease)).tw,kf. | 6305 |
| 12 | 6 or 7 or 8 or 9 or 10 or 11 | 202034 |
| 13 | 5 and 12 | 13542 |
| 14 | exp Diet/ | 362170 |
| 15 | exp nutrition/ | 2414395 |
| 16 | exp Food/ | 1136033 |
| 17 | (diet? or dietary or nutrition* or nutrient* or food? or feed or feeding).tw,kf. | 1773452 |
| 18 | (fasts or fasting or weight or obes* or BMI or body mass index or quetelet or body fat).tw,kf. | 1932806 |
| 19 | *body mass/ | 36870 |
| 20 | exp *body weight/ | 87111 |
| 21 | (supplement? or vitamin? or mineral? or carbohydrate? or lipid?).tw,kf. | 1342602 |
| 22 | microbiome/ or microflora/ or bacterial microbiome/ | 49880 |
| 23 | ((gut or gastr* or intestin*) adj3 (microbio* or microflora or flora)).tw,kf. | 73816 |
| 24 | 14 or 15 or 16 or 17 or 18 or 19 or 20 or 21 or 22 or 23 | 5286397 |
| 25 | 13 and 24 | 2971 |

**Table S5: Search strategies for Cochrane Central Register of Controlled Trials (Ovid)**

Database: EBM Reviews - Cochrane Central Register of Controlled Trials January 2022

| **#** | **Searches** | **Results** |
| --- | --- | --- |
| 1 | inflammatory bowel diseases/ or crohn disease/ or ileitis/ | 2169 |
| 2 | Crohn*.tw,kw. | 5499 |
| 3 | ("Inflammatory bowel disease*" or IBD).tw,kw. | 4369 |
| 4 | ((regional* or terminal) adj3 (ileiti* or enteritis or enterocolitis)).tw,kw. | 64 |
| 5 | 1 or 2 or 3 or 4 | 8289 |
| 6 | Constriction, Pathologic/ | 844 |
| 7 | Intestinal Obstruction/ | 351 |
| 8 | Fibrosis/ | 1714 |
| 9 | ((gut or colon or colonic or ileal or ileum or intestin* or duodenal or duodenum or jujenal or jujenum or abdominal or "intra-abdominal" or anastomotic) adj5 (strictur* or constriction* or stenos* or atresia* or obstruct* or narrowing or lesion* or fibros* or abscess* or lesion* or "wall thick*" or perfusion* or dilation)).tw,kw. | 4414 |
| 10 | (bowel adj5 (strictur* or constriction* or stenos* or atresia* or obstruct* or narrowing or lesion* or fibros* or abscess* or lesion* or "wall thick*" or perfusion* or dilation)).tw,kw. | 1150 |
| 11 | (strictur* adj3 (lesion* or fibrotic or inflammatory or complication* or disease)).tw,kw. | 341 |
| 12 | 6 or 7 or 8 or 9 or 10 or 11 | 7925 |
| 13 | 5 and 12 | 477 |
| 14 | exp Diet/ | 18959 |
| 15 | enteral nutrition/ or nutrition therapy/ or exp diet therapy/ or nutritional support/ or exp Parenteral Nutrition/ | 9411 |
| 16 | exp Food/ | 52820 |
| 17 | (diet? or dietary or nutrition* or nutrient* or food? or feed or feeding).tw,kw. | 149087 |
| 18 | (fasts or fasting or weight or obes* or BMI or body mass index or quetelet or body fat).tw,kw. | 194276 |
| 19 | body mass index/ | 10926 |
| 20 | exp body weight/ | 29697 |
| 21 | (supplement? or vitamin? or mineral? or carbohydrate? or lipid?).tw,kw. | 113010 |
| 22 | microbiota/ or gastrointestinal microbiome/ | 1084 |
| 23 | ((gut or gastr* or intestin*) adj3 (microbio* or microflora or flora)).tw,kw. | 6263 |
| 24 | 14 or 15 or 16 or 17 or 18 or 19 or 20 or 21 or 22 or 23 | 355675 |
| 25 | 13 and 24 | 114 |

**Table S6: Search strategies for CINAHL (EBSCO)**

Database: CINAHL Plus with full-text

| **#** | **Query** | **Results** |
| --- | --- | --- |
| S1 | (MH "Inflammatory Bowel Diseases") OR (MH "Crohn Disease") | 14,200 |
| S2 | TI Crohn* OR AB Crohn* | 8,543 |
| S3 | TI ( "Inflammatory bowel disease*" or IBD ) OR AB ( "Inflammatory bowel disease*" or IBD ) | 11,297 |
| S4 | TI ( ((regional* or terminal) N4 (ileiti* or enteritis or enterocolitis)) ) OR AB ( ((regional* or terminal) N4 (ileiti* or enteritis or enterocolitis)) ) | 71 |
| S5 | S1 OR S2 OR S3 OR S4 | 19,399 |
| S6 | (MH "Constriction, Pathologic") | 5,031 |
| S7 | (MH "Duodenal Obstruction") OR (MH "Intestinal Obstruction") | 4,658 |
| S8 | (MH "Fibrosis") | 7,613 |
| S9 | TI ( ((gut or colon or colonic or ileal or ileum or intestin* or duodenal or duodenum or jujenal or jujenum or abdominal or "intra-abdominal" or anastomotic) N6 (strictur* or constriction* or stenos* or atresia* or obstruct* or narrowing or lesion* or fibros* or inflamm* or abscess* or lesion* or "wall thick*" or perfusion*)) ) OR AB ( ((gut or colon or colonic or ileal or ileum or intestin* or duodenal or duodenum or jujenal or jujenum or abdominal or "intra-abdominal" or anastomotic) N6 (strictur* or constriction* or stenos* or atresia* or obstruct* or narrowing or lesion* or fibros* or inflamm* or abscess* or lesion* or "wall thick*" or perfusion*)) ) | 13,536 |
| S10 | TI ( (bowel N6 (strictur* or constriction* or stenos* or atresia* or obstruct* or narrowing or lesion* or fibros* or inflammation or abscess* or lesion* or "wall thick*" or perfusion*)) ) OR AB ( (bowel N6 (strictur* or constriction* or stenos* or atresia* or obstruct* or narrowing or lesion* or fibros* or inflammation or abscess* or lesion* or "wall thick*" or perfusion*)) ) | 4,224 |
| S11 | TI ( (stricturing lesion* or fibrotic stricture* or inflammatory stricture*) ) OR AB ( (stricturing lesion* or fibrotic stricture* or inflammatory stricture*) ) | 350 |
| S12 | S6 OR S7 OR S8 OR S9 OR S10 OR S11 | 30,711 |
| S13 | S5 AND S12 | 2,835 |
| S14 | (MH "Body Mass Index") OR (MH "Body Weight") OR (MH "Body Weight Changes") OR (MH "Diet+") OR (MH "Nutrition") | 251,930 |
| S15 | (MH "Nutritional Support+") | 42,071 |
| S16 | (MH "Diet Therapy+") | 35,777 |
| S17 | (MH "Food+") | 194,378 |
| S18 | (MH "Gut Microbiota") OR (MH "Microbiota") | 6,290 |
| S19 | TI ( (diet# or dietary or nutrition* or nutrient* or food# or feed or feeding) ) OR AB ( (diet# or dietary or nutrition* or nutrient* or food# or feed or feeding) ) | 333,574 |
| S20 | TI ( (fasts or fasting or weight or obes* or BMI or body mass index or quetelet or body fat) ) OR AB ( (fasts or fasting or weight or obes* or BMI or body mass index or quetelet or body fat) ) | 354,553 |
| S21 | TI ( (supplement# or vitamin# or mineral# or carbohydrate# or lipid#) ) OR AB ( (supplement# or vitamin# or mineral# or carbohydrate# or lipid#) ) | 153,670 |
| S22 | TI ( ((gut or gastr* or intestin*) N4 (microbio* or microflora or flora)) ) OR AB ( ((gut or gastr* or intestin*) N4 (microbio* or microflora or flora)) ) | 8,806 |
| S23 | S14 OR S15 OR S16 OR S17 OR S18 OR S19 OR S20 OR S21 OR S22 | 840,728 |
| S24 | S13 AND S23 | 927 |
| S25 | S13 AND S23  Limit Source Types: Academic Journals | 879 |
