# Supplementary material for: Nutritional interventions in adult fibrostenotic Crohn’s disease: A systematic review
Source: Front Nutr. 2023 Feb 21;10:1017382. doi: 10.3389/fnut.2023.1017382 (PMC9988909; doi:10.3389/fnut.2023.1017382)
Supplement: Supplementary file 2 [file Table_2.DOCX]

**Table S1. Quality Assessment of Observational Studies for Dietary Interventions in Fibrostenotic Crohn’s Disease using the Newcastle–Ottawa scale. The maximum number of stars each study can receive is 9 (maximum 4 for selection, 2 for comparison and 3 for outcome.**

| **Author, year** | **Selection** | **Comparability** | **Outcome** | **Score** |
| --- | --- | --- | --- | --- |
| Hu, 2014 | *0** | *0 | *0* | 6 |
| Marafini, 2020 | **** | ** | *0* | 8 |
| Ostro, 1985 | *0** | 00 | *** | 6 |
| Teahon, 1990 | **** | *0 | 0*0 | 6 |
| Yang, 2015 | **** | *0 | *0* | 7 |

**Table S2. GRADE Assessment of Studies for Dietary Interventions in Fibrostenotic Crohn’s Disease**

| **Study** | **Starting Level of Evidence** | **Reasons for Decreasing the Level of Evidence** | | | | | **Reasons to increase level of evidence (strong association, plausible confounding and bias adjustment)** | **Final Level of Evidence** |
| --- | --- | --- | --- | --- | --- | --- | --- | --- |
|  |  | **Risk of Bias** | **Inconsistency** | **Indirectness** | **Imprecision** | **Publication Bias** |  |  |
| **Observational trials - 5** | Low | Yes | Yes | No | Yes | No | Low Number of Studies | Very Low |
